# Potential Benefit of Selective CMV Testing after Failed Newborn Hearing Screening

**DOI:** 10.3390/ijns4020020

**Published:** 2018-06-19

**Authors:** Peter Kummer, Steven C. Marcrum

**Affiliations:** 1 Section Phoniatrics and Pediatric Audiology, Department of Otolaryngology, University Hospital Regensburg, Franz-Josef-Strauß-Allee 11, 93053 Regensburg, Germany; 2 Department of Otolaryngology, University Hospital Regensburg, Franz-Josef-Strauß-Allee 11, 93053 Regensburg, Germany

**Keywords:** congenital cytomegalovirus, sensorineural hearing loss, newborn hearing screening

## Abstract

Evidence-based guidelines for the prevention, diagnosis and treatment of congenital cytomegalovirus (cCMV) were recently released by two independent expert groups. Of particular emphasis was the relationship between cCMV and sensorineural hearing loss (SNHL), a major component of the virus’ overall disease burden. In this study, a literature review was performed to estimate the proportion of cCMV-related SNHL cases, which might be identified through selective cCMV testing following failed newborn hearing screening. Furthermore, it was of interest to estimate the potential benefit of emerging antiviral therapies. Currently, at most 10% of cCMV-related SNHL is likely to be identified clinically. Through use of a selective cCMV testing protocol, however, a significant improvement in the identification rate can be achieved. Recent expert group statements strongly recommend antiviral therapy in cases of moderate-to-severe disease, especially in the presence of central nervous system involvement. Though differences exist between recommendations in instances of isolated SNHL or SNHL in combination with only mild symptoms, the majority of experts in both groups offered at least a weak recommendation for antiviral treatment. Available results suggest antiviral treatment could therefore benefit a meaningful proportion of newborns referred for cCMV testing following failed newborn hearing screening.

## 1. Introduction

Congenital cytomegalovirus (cCMV) is the leading non-genetic cause of sensorineural hearing loss (SNHL) worldwide and a significant contributor to neurodevelopmental delay [1,2,3,4]. Whereas all genetic causes taken together account for 50–60% of congenital SNHL, cCMV alone is thought to be responsible for 1 out of 5 cases of SNHL at birth and 1 out of 4 cases of SNHL at 4 years of age [2,3,4,5]. Therefore, cCMV can be described as a major cause of not only congenital, but also delayed-onset SNHL.

Early identification of cCMV infection has the potential to significantly impact the treatment of permanent childhood hearing loss (PCHL), which has for several decades been shaped predominantly by an early intervention focus (i.e., treatment with hearing aids or cochlear implants and the provision of intervention/developmental services). Indeed, the worldwide spread of universal newborn hearing screening (UNHS) programs has been driven in large part by the success the programs have had in mitigating many of the negative effects of PCHL on children’s speech, language and literacy development [6,7,8,9,10,11]. However, with the advent of efficient means of diagnosing cCMV infection, an additional option of antiviral therapy for congenital CMV-related SNHL has arisen. This option represents a distinct contribution to the treatment of PCHL. For example, good evidence from one randomised controlled trial (RCT) indicated that antiviral therapy with intravenous ganciclovir (GCV) over a period of 6 weeks significantly reduced future SNHL when given to babies born with symptoms of cCMV infection [12]. In a second RCT [13], oral valganciclovir (VGCV), provided for a period of over 6 months, was shown to improve hearing and developmental outcomes more modestly in the longer term, but demonstrated the additional advantages that it was not associated with an excessive risk of neutropenia and avoided the need to maintain intravenous access for prolonged periods of time.

Two informal expert groups, one comprised of North American, Australian, and British specialists in the area of CMV (ICCRG—International Congenital Cytomegalovirus Recommendations Group [14], and the other of European experts (ESPID—European Society of Paediatric Infectious Diseases [15], convened at conferences in 2015 and started the work of reviewing and grading available evidence in an effort to highlight both points of controversy and consensus in cCMV testing and treatment. These efforts resulted in the generation of recommendations for the diagnosis, prevention, and therapy of cCMV. Statements from the two groups were recently published [14,15], which has led national guideline development committees, such as those in Germany [16,17], to begin translating recommendations into clinical practice. Specifically, these committees have focused great attention on early antiviral therapy in groups of infants, well-defined in terms of both clinical symptomatology and disease severity.

However, only approximately 1 out of 8 infected newborns is symptomatic at birth [1,18,19] and symptoms, when present, may be unspecific or otherwise insufficient to trigger prompt CMV testing [20]. Accurate diagnosis, on the other hand, requires virus isolation or polymerase chain reaction (PCR) of CMV in urine or saliva within the first three weeks of life [21]. Tests taken after this time no longer clearly distinguish between congenital and acquired forms of the infection, a distinction which is critical for identifying associated birth defects or predicting developmental disabilities [20,22,23,24]

Studies are beginning to show that real-time polymerase chain reaction (PCR) assays, with both sensitivities and specificities amenable to mass screening [25], are efficacious, feasible and cost-effective for the purpose of cCMV screening [26,27,28]. Given this, two recommendations for cCMV screening implementation have gained significant attention. The first involves limiting screening to those newborns who have already failed a newborn hearing screening, while the second constitutes a universal screening [29,30,31,32,33].

It was the objective of this article to review the epidemiology of congenital cytomegalovirus infection and its effects in relation to hearing loss, present the potential benefits and drawbacks of targeted cCMV testing, as well as assess the efficacy of selective testing and the potential benefit of additional antiviral therapy according to recently published recommendations.

## 2. Epidemiology, Classification and Diagnosis of Congenital CMV

Results from universal CMV screening programs in developed countries suggest the mean prevalence of congenital cytomegalovirus infection to be approximately 0.58% (95% CI: 0.41–0.79), though prevalence has been shown to vary meaningfully across populations according to numerous factors [2]. For example, Dollard et al. [1] reported the prevalence of congenital CMV infection in infants born into families with low socioeconomic status (mean: 1.2%; Range: 0.9–1.3%) to be more than 300% that of infants born into families with middle socioeconomic status (mean: 0.39%; Range: 0.3–0.5%), on average. Unfortunately, no representative datasets exist currently for babies born in Germany, though prevalence estimates of 0.2–0.6% have been reported [16,34].

For the purposes of this study, the 0.6% cCMV prevalence estimate of Cannon et al. [20], which was based on the results of a comprehensive literature review [1], was used. Studies contributing to that estimate enrolled at least 800 children from high-income countries and identified their samples through universal screening at birth with viral culture or PCR detection methods. 

The clinical spectrum of cCMV infection ranges from the fully asymptomatic (ca. 85–90%) all the way to life-threatening illness [18,19]. For the majority of cCMV-positive infants, symptoms of the cCMV infection are discrete and cannot be readily clinically diagnosed. Whereas disseminated petechiae, thrombocytopenia, hepatosplenomegaly, and hepatitis (raised transaminases or bilirubin) are characteristic symptoms of congenital CMV-infection, intrauterine growth restriction, central nervous system involvement (in the form of microcephaly or radiographic abnormalities such as ventriculomegaly, intracerebral calcifications, periventricular echogenicity, cortical or cerebellar malformations), chorioretinitis, sensorineural hearing loss and premature birth are not pathognomonic for congenital CMV infection and exhibit significant variability [20].

Accepted definitions of symptomatic, congenital CMV infection therefore frequently include symptoms that generally do not prompt the physician to order a CMV test. In the presence of known CMV-related signs or symptoms, current clinical guidelines [16,17] recommend a full laboratory workup. At minimum, this workup should include a quantitative analysis of CMV-DNA within a saliva sample as soon as possible after birth, with cCMV positive results being confirmed via CMV-PCR analysis of a urine sample. Unfortunately, clinical follow through is commonly sporadic.

The proportion of symptomatic children which might be diagnosed clinically was recently estimated [20] by comparing numbers of symptomatic children from studies with active surveillance in well-defined populations with prevalence numbers from studies with newborn CMV screening, with results ranging from 3.8% to 25.0%. In agreement with these estimates, calculations from screened populations also showed that only 13% of newborns with symptomatic cCMV had more than one symptom [35]. A conservative estimate of 25% of symptomatic children, which would be diagnosed clinically, (i.e., tested for CMV because of their presenting clinical signs or symptoms) was therefore adopted [20] and is also used for the purposes of this study (see Table 1). What is troubling, this result indicates that 75% of symptomatic children will currently not be diagnosed without targeted or universal screening measures. Of the approximately 6000 children with cCMV per 1,000,000 births, a correct CMV diagnosis, based solely on apparent clinical symptoms, is possible in only approximately 3.2% of cases. 9.6% and 87.2% will be missed due to nonspecific symptoms and the complete lack of symptoms, respectively (see Table 1).

## 3. Characteristics of cCMV-Related Hearing Loss

According to a meta-analysis of studies investigating universal CMV-screened children [2], sensorineural hearing loss occurs in approximately 12.6% of cCMV infections (95% CI: 10.2–16.5), or 1 out of 3 symptomatic children and 1 out of 10 asymptomatic children (95% CI: 23.2–43.2 and 6.3–14.3, respectively). There is no cCMV-pathognomonic configuration for hearing loss. Characteristic of both symptomatic and asymptomatic cases, however, is the variable nature of hearing function, with fluctuating thresholds (present for 20% and 24% of cases for the two groups, respectively), late onset [present for 18% (95% CI: 5.9–36.2) and 9% (95% CI: 0.8–24.5)] and progressive hearing loss [18% (95% CI: 3.5–39.4) and 20% (95% CI: 5.3–41.8)] observable for meaningful proportions of infected infants. Significant heterogeneity, however, was found between studies in terms of the reported prevalence of late onset, progressive hearing loss and fluctuations, especially for older studies conducted before the widespread establishment of newborn hearing screening programs [2]. Among symptomatic children, the majority exhibited bilateral hearing loss (71.2%, 95% CI, 64.2–77.8) which was generally severe to profound in degree. Among asymptomatic children, unilateral loss predominated (56.9%, 95% CI: 41.4–71.8), which was also mostly severe to profound in degree and often represented the only significant consequence of the infection.

In an effort to estimate the disease burden of cCMV-associated hearing loss, epidemiological data [36,37] obtained from Cannon et al. [20] were tabulated (see Table 1). These findings describe not only the prevalence, but also the time course of hearing loss in symptomatic and asymptomatic children. This delineation allows not only for estimating the number of cases of cCMV-associated hearing loss within the first 6 years of life per 1 million births, but also the proportion of those children, which, due to clinical symptomatology or selective CMV testing, can receive a definitive cCMV diagnosis and subsequent directed antiviral therapy, or which, in the absence of clinical symptomatic or selective CMV testing, will not be diagnosed or treated.

Independent of the absolute prevalence of cCMV, however, it remains that hearing loss will largely be missed unless the infection is identified during pregnancy, by chance or by more or less systematic screening (see Table 1). Only 1 of 8 children presents signs of infection that are clinically apparent. Estimated conservatively, at most 1 out of 4 of these symptomatic children is likely to be diagnosed clinically (i.e., because of presenting symptoms whose combination is typical enough to trigger testing for CMV infection) [20]. Thus, no more than 10% (53/503) of children with cCMV related hearing loss at birth are diagnosed due to unambiguous clinical appearance. At least 31% (158 of 503) present symptoms that do not prompt testing. The majority (58%, 293 of 503 cases) of SNHL cases will be found among the asymptomatic children, who do not present any clinically visible symptoms.

## 4. Selective cCMV Testing after Failed Newborn Hearing Screening

The benefit that might result from different implementations of CMV testing or screening may be estimated from the data in Table 1. Approximately half (52% = 503/971) of all instances of cCMV-associated hearing loss occurring during the first 6 years of life are present from birth. Targeted CMV testing among newborns who fail hearing screening tests thus has been explored by different groups [38,39,40,41]. Both the terms “selective testing” and “selective screening” have been used to describe the above procedure. However, experts of a German guidelines development group [16] have joined others [42] in designating this procedure “selective testing”, thereby highlighting the diagnostic rather than screening character of this testing in cases of reasonable suspicion of congenital hearing loss after failed newborn hearing screening.

The selective testing approach was first described in a large-scale, retrospective study in the Southwestern United States [38]. Over a 5-year period, 5% of infants (24/483) who did not pass the AABR-based newborn hearing screen, and subsequently underwent CMV testing, and 6% (16/256) of those infants with subsequently confirmed hearing impairment, tested positive for CMV. Of those 16 infants, 12 (75%) were identified as having cCMV infection only because of a failure to pass newborn hearing screening tests. From a statewide NHS program in Utah, 62% of cases recently underwent CMV screening only after failing hearing screening [41]. Similarly, 14 of these 234 (6%) infants tested within 21 days were CMV positive. Both studies thus succeeded in identifying cCMV in 5–6% of congenital SNHL. Considering that a portion of the cases may be missed [26], this might correspond to a proportion of about 10% of congenital SNHL due to cCMV. From a retrospective study in Italy that estimated the impact of cCMV in 130 children with SNHL >40 dB by detection of CMV DNA in stored samples of neonatal dried blood, 10% (9 of 87) of SNHL cases diagnosed within the first 2 months of life were attributed to cCMV, while 34.2% (13 of 38) of SNHL cases diagnosed in early childhood were attributed to CMV [5]. These data are in agreement with other estimates, which identify cCMV as the cause of approximately 20% of cases of congenital SNHL and 25% of cases at the age of 4 years [3,4].

The proportion of newborns which did not pass NHS and which might be identified with cCMV-related congenital SNHL was recently evaluated in a multicenter diagnostic study [26]. In that study, 443 newborns that were diagnosed with cCMV after the universal cCMV screening of 99,945 newborns underwent diagnostic audiological examinations at 3 to 8 weeks of age. Test measures included tone-burst auditory brainstem response thresholds, distortion product otoacoustic emissions, bone-conduction measurements, tympanometry and acoustic reflex testing. Newborn hearing screening, most commonly a 2-stage protocol in the hospital and an additional outpatient hearing screening, could identify only 57% of infants with congenital cCMV-related hearing loss—47% of asymptomatic and 69% of symptomatic cCMV cases. However, 43% of all newborns with cCMV-related SNHL were not identified via the NHS. This lack of sensitivity has multiple causes. First, 60% of those newborns missed exhibited mild hearing loss, which is notoriously difficult to identify via 2-stage NHS protocols designed to identify ears with at least moderate hearing loss [43,44]. Second, the nature of cCMV-related SNHL, with its potential for progression and/or late-onset [2,45], suggests that hearing losses might have been absent or mild at the time of testing, despite being moderate by 8 weeks of age.

From that, selective CMV testing of newborns who do not pass NHS could be used to identify the majority of CMV-related SNHL at birth (see Table 1), i.e., about 27% of cases that occur in asymptomatic cCMV (47% × 293/503) and 29% in symptomatic cCMV (69% × (53 + 158)/503), independent of clinical symptoms. This represents a meaningful improvement over the at most 10% (53/503) of symptomatic cCMV cases likely to be clinically identified [20].

## 5. CMV Screening Tests

Comprehensive studies have evaluated which methods are most appropriate for laboratory diagnosis of cCMV-infection in the context of a mass screening (i.e., both in selective testing and universal screening). Since dried blood spots (DBS) are often routinely collected for metabolic screening, there has been interest in PCR–based methods for CMV screening. A multicenter study, however, that compared single-primer PCR and 2-primer real-time PCR analysis of DBS to saliva rapid culture, demonstrated low sensitivity for use in mass screening for cCMV in 20,448 infants [46]. Only 17 of 60 (28%) and 11 of 32 (34%) cases were identified as positive when using both methods, yielding a sensitivity of 28.3% (95% CI: 17.4–41.4%) and 34.4% (95% CI: 18.6–53.2%). It was concluded that approximately two-thirds of infections were missed using this method, limiting its value as a screening test. The sensitivity of PCR in detecting CMV on DBS, however, was much lower than in other studies. In a meta-analysis, sensitivity of around 84% was found, though it is known to be affected by factors such as laboratory technique, extraction method, and the population being tested [47,48,49]. However, none of these methods were evaluated in screening of unselected neonates. A positive DBS CMV PCR taken in the first 3 weeks of life may confirm the diagnosis of cCMV, though a negative result cannot reliably exclude cCMV. Diagnosing cCMV-infection after the first 21 days of life, however, can only be achieved via PCR testing of DBS samples taken during newborn screening within the first three days of life.

Both urine and saliva from infected newborns contain high CMV viral loads and are amenable to rapid testing by PCR with high sensitivity [21]. However, testing should be performed as early as possible (no later than 3 weeks of age), as diagnostic tests performed after this time no longer distinguish congenital infection from a postnatal cytomegalovirus infection, which does not cause hearing loss [50,51]. Likewise, to avoid false-positive findings through CMV contamination via breast milk, saliva samples should be taken immediately before feeding in breastfed newborns [52]. CMV positive saliva samples should be confirmed with urine, as false-positive results have been reported [21,25,52,53]. Urine collection, however, is time-consuming and practically difficult [14,16]. Samples could not be analyzed in one third of the cases in a study comparing diagnostic accuracy between saliva and urine PCR [46]. Saliva samples, on the other hand, are noninvasive, easy to obtain in comparison to urine samples and can be stored and transported at room temperature offering obvious advantages over urine in their potential use as a screening tool.

In a large, prospective, multicenter screening study of newborns, real-time PCR assays of liquid-saliva and dried-saliva specimens were compared with rapid culture of saliva specimens obtained at birth [25]. Of 17,662 newborns screened with the liquid-saliva PCR assay, 85 infants (0.5%; 95% CI: 0.4 to 0.6) had positive results on both rapid culture and PCR assay. The sensitivity and specificity of the liquid-saliva PCR assays were 100% (95% CI: 95.8 to 100) and 99.9% (95% CI: 99.9 to 100), respectively. With the dried-saliva PCR assay, 74 of 17,327 newborns were positive for CMV, whereas 76 were found to be CMV-positive on rapid culture. Sensitivity and specificity of the dried-saliva PCR assay were therefore 97.4% (95% CI: 90.8 to 99.7) and 99.9% (95% CI: 99.9 to 100), respectively. False positive results that can cause considerable anxiety for parents were less than 0.03% of both liquid and dried saliva.

Both recent expert group statements concurred in strongly recommending that diagnosis of cCMV infection in neonates include real-time PCR of saliva, urine, or both (as soon as possible after birth, but within the first 3 weeks of life). Furthermore, saliva was identified as the preferred sample, with a recommendation of urine to be used for confirmation of a positive result [14,15].

## 6. Benefit of Antiviral Therapy

The benefit of selective CMV testing with respect to hearing loss arises primarily from the positive effects of antiviral therapy on auditory thresholds and the associated improvements in performance in both aided and unaided conditions. Additional benefits come in the form of early intervention services and increased awareness on the part of caregivers and educators. However, the potential benefits of antiviral therapy must be viewed in relation to the potential risks, namely toxicities. Side effects and associated risks of ganciclovir (GCV) and valganciclovir (VGCV), such as neutropenia, are well-known [12,13,54], although the risk/benefit analysis has changed with the availability of VGCV liquid formulation [13,55]. Possible risks, such as gonadal dysgenesis and carcinogenicity have, to date, only been observed in animal models [56,57]. No antiviral drugs are currently licensed for the management of cCMV infection in humans.

Despite many case reports and cohort studies, data from only 2 RCTs on antiviral treatment for cCMV have been published [12,13,58,59,60,61,62,63,64,65,66,67,68,69]. A phase III RCT, primarily assessing the hearing outcomes of newborns with cCMV, was conducted by the National Institute of Allergy and Infectious Diseases Collaborative Antiviral Study Group (CASG) [12]. Over a 10 year period, the study recruited 100 newborns (<1 month of age) with gestational age ≥32 weeks and weight ≥1200 g at birth, and with clinically apparent infection and evidence of CNS disease (including microcephaly, intracranial calcification, abnormal CSF indices for age, hearing deficit and chorioretinitis). The infants were randomized to receive 6 weeks of intravenous GCV 6 mg/kg/dose every 12 h or no treatment. Although there was significant loss to follow-up, improved hearing and neurodevelopmental outcomes could be identified. Specifically, between baseline and 6 months, 21 of 25 (84%) GCV recipients had improved hearing or maintained normal hearing versus 10 of 17 (59%) control patients. None of the 25 GCV recipients had worsened hearing thresholds versus 7 of 17 (41%) control patients *(p* < 0.01). At 1 year and beyond (i.e., at an average age of about 2 years), 5 of 24 (21%) GCV recipients had worsened hearing versus 13 of 19 (68%) control patients. It was concluded that GCV prevents hearing deterioration at 6 months and may prevent hearing deterioration at more than 1 year.

More recently, a second randomized trial conducted by the CASG compared antiviral efficacy and safety of a 6-week versus 6-month treatment with oral VGCV [13], thus decreasing some of the risks associated with GCV (i.e., risks associated with the presence of an indwelling central venous catheter or of neutropenia) that had been significantly associated with treatment. In this trial, babies with any evidence of symptomatic disease (including non-CNS) with age <1 month, gestational age ≥32 weeks and weight ≥1800 g at birth were enrolled. A modest benefit on both 2-year hearing and neurodevelopmental outcomes was shown for the 6-month treatment course. Although best-ear hearing at 6 months was similar in both groups, total-ear hearing (hearing in one or both ears that could be evaluated) was more likely to be improved or to remain normal at 12 months in the 6-month treatment group than in the 6-week treatment group (73% vs. 57%). Further, the benefit remained after 24 months (77% vs. 64%). This advantage for the longer therapy duration was statistically significant, but only when adjusted for baseline CNS involvement. At 24 months, the 6-month treatment group had better neurodevelopmental scores on both the language-composite component and on the receptive-communication scale of the Bayley Scales of Infant and Toddler Development. It was concluded that treating symptomatic congenital CMV infection with VGCV for 6 months, as compared with 6 weeks, did not improve hearing in the short term, but appeared to improve hearing and developmental outcomes modestly in the longer term. Significant neutropenia, however, was less commonly reported with VGCV (21% compared with 65%) [12,13,54,60]. Additionally, no increased toxicity was observed in those children randomized after 6 weeks to receive 6-month treatment compared with placebo.

### 6.1. Recommendations for Early Antiviral Therapy in Symptomatic CMV Infection

Clinical trials provide solid evidence upon which to base treatment decisions for only a portion of the infants presenting to clinicians. Based on the above-mentioned studies, which were rated as level 1 evidence according to the Oxford Centre for Evidence Based Medicine (CEBM), the International Congenital Cytomegalovirus Recommendations Group (ICCRG [14]) recommended oral VGCV within the first month of life for a duration of 6 months in congenitally-infected neonates with multiple manifestations of moderate-to-severe symptomatic disease [13]. Manifestations might include thrombocytopenia, petechiae, hepatomegaly, splenomegaly, intrauterine growth restriction, hepatitis (raised transaminases or bilirubin), or central nervous system involvement (i.e., microcephaly, radiographic abnormalities consistent with cytomegalovirus central nervous system disease, ventriculomegaly, intracerebral calcifications, periventricular echogenicity, cortical or cerebellar malformations), abnormal cerebrospinal fluid indices for age, chorioretinitis, sensorineural hearing loss, or the detection of cytomegalovirus DNA in cerebrospinal fluid. For the purpose of improving audiological and developmental outcomes, treatment should not exceed 6 months. Neonates with asymptomatic congenital cytomegalovirus infection should not be given antiviral therapy (level 3 evidence).

Using the GRADE rating system, the ESPID expert group recommended [15] that babies with evidence of CNS disease (Quality A, Strength 1) receive oral VGCV as the drug of choice (Quality A, Strength 1) for a period of 6 months (Quality B, Strength 2). Additionally, it was recommended that children with life-threatening disease or severe single- or multi-organ disease should receive treatment (Quality B, Strength 1). However, the group recommended against treating asymptomatic children with no clinical/laboratory findings, as current evidence does not support treatment in these patients (Quality D, Strength 1). 

In babies with one or two mild, transient, or clinically insignificant manifestations of cCMV infection (e.g., mild hepatomegaly or a single measurement of low platelet count or raised levels of alanine aminotransferase), the ICCRG recommended against the routine use of antiviral therapy [14]. For the ESPID group, a majority weakly recommended against treatment with antivirals (Quality B, Strength 2) [15].

If it is assumed that cases of moderate-to-severe, symptomatic disease can be clinically diagnosed, recommendations for antiviral therapy will apply to about 10% (53/503) of cCMV-related SNHL cases at birth (see Table 1) and may comprise, even if all symptomatic cases were included, at most 42% ((53 + 158)/503) of cCMV-related, congenital SNHL cases. Approximately 58% (293/503) of cases, which occur in asymptomatic newborns, however, will be excluded when indication for antiviral therapy is based solely on the severity of the clinical symptomatology or CNS involvement. It is critical therefore, for both the indication for antiviral therapy and the potential benefit of selective testing to be considered in light of anticipated effects on degree of sensorineural hearing loss—even if occurring in isolation—independent of the severity of the overall symptomatology.

### 6.2. Recommendations for Early Antiviral Therapy in Isolated Sensorineural Hearing Loss

In cases of asymptomatic, congenital cytomegalovirus infection with isolated sensorineural hearing loss, the ICCRG recommended against routine treatment with VGCV due to a lack of quality evidence (level 3 evidence). However, as only one infant with isolated sensorineural hearing loss had been enrolled in a RCT [13], it was suggested that patient data be accumulated in an effort to improve understanding of the safety of such an approach for these infants.

A similar argument was made by the ESPID expert group [15]. In essence, the controversy was centered on whether isolated SNHL should be considered a CNS manifestation of infection and, as a consequence, whether such children should be considered comparable to those with CNS disease included in published clinical trials [15]. It was stated that the exact pathophysiology of SNHL was not clear, but that it was likely secondary to infection and degradation of sensory structures within the inner ear [2,70]. No published studies have as yet addressed this specific population. A nonrandomized cohort study, however, is in progress, which is investigating whether early treatment of infants up to 12 weeks of age with oral VGCV can halt progression of hearing loss (clinicaltrials.gov NCT02005822). Though no consensus could be reached due to the large range of hearing losses encountered, a majority of the ESPID experts concluded that babies with isolated, confirmed SNHL should indeed be categorized within the “severe” CNS group. As the main treatment benefit was a preservation, rather than improvement, of hearing thresholds, a majority of the experts also recommended inclusion of SNHL at birth within their indications for a 6-week to 6-month antiviral treatment regimen. The relatively low quality of evidence supporting this recommendation should be considered when contemplating clinical implementation of this specific recommendation (Grade C, Strength 1).

Whereas the ICCRG recommended that decisions about the treatment of isolated sensorineural hearing loss be made on a case-by-case basis, a majority the ESPID expert group members recommended the addition of SNHL at birth to the indications for treatment. If SNHL was included in the indications for antiviral treatment irrespective of severity of additional clinical symptoms, recommendations of antiviral treatment would apply to all cases of cCMV-related congenital hearing loss—constituting about half of the disease burden of hearing loss. The potential benefit of selective testing, which may allow detection of up to 57% of cases with congenital SNHL, would therefore increase considerably.

### 6.3. Recommendations for Later Antiviral Therapy

Almost half of the hearing loss-related disease burden accrued within the first 6 years of life exists not from birth, but rather occurs with delayed onset (468/971; see Table 1). Independent of how cCMV is detected, be it via universal screening or selective testing, audiological follow up will be necessary to detect the onset of hearing loss in a large proportion of cases. Testing at 3 to 6-month intervals within the first year of life [15], 6-month intervals until 3 years of age [14,15], and 12-month intervals until 6 years of age [15] or through adolescence (ages 10–19) [14] have been recommended even when no hearing loss is detected at birth in cCMV-positive infants.

With average universal newborn hearing screening refer-rates of 5.3%, as shown by an evaluation of the German newborn hearing screening system from 2011 to 2012 [71], cCMV testing within a selective program would be required for 1 out of 20 infants. In exchange for this increased diagnostic burden, in combination with appropriate follow-up, the early detection of cCMV-related SNHL would even be possible in cases with delayed onset. This is of great importance, as delayed-onset hearing loss comprises fully 46% of all cCMV-related SNHL (see Table 1). Given a 5% refer-rate in NHS, these cases may comprise 2.3% (5% × (65 + 382)/971) of these cases. It should be borne in mind, however, that definitive hearing status diagnostics are not always performed within the first 4 weeks of life. A question remains as to whether a start of antiviral therapy should be recommended even outside the 4-week window of evidence, despite its being set as the cut-off in both RCTs [12,13].

The highest quality evidence available is limited to retrospective case series of small numbers of babies treated outside the newborn period. Amir et al. [68] reported on 21 children, the majority of which had asymptomatic cCMV or only lenticulostriatal vasculopathy, which were treated with antivirals over a 9 to 12 month period starting at a median age of 8 months. At the last follow-up (median age of 24 months), hearing impairment improved in 29 of 35 ears evaluated. For ears with initially mild to moderate impairment, final results were commonly within normal limits. Furthermore, no evidence of hearing loss progression could be identified. Del Rosal et al. [67] reported on 13 cases with CNS involvement who received either oral VGCV or oral VGCV in combination with intravenous GCV at a median age of 3 months. Eleven children (85%) had hearing defects at baseline, compared to 50% at 12 months. However, although 7 out of 10 ears with mild to moderate hearing loss improved, only 1 out of 8 with severe hearing loss showed meaningful improvement. Finally, no deterioration was found in 8 initially normal hearing ears at 12 months.

Both ICCRG and ESPID expert groups state that treatment of older children has not been addressed in any RCTs [14,15], although it is acknowledged that the 28-day cutoff is also not evidence-based [15]. Two promising trials, however, were started in 2015 and 2017. Researchers for the first study (NCT02606266) are recruiting participants for a randomized, controlled phase 2 trial of VGCV therapy in children up to 4 years of age with congenital cytomegalovirus infection and hearing loss. The second study (NCT01649869) is a randomised efficacy study to evaluate the benefit of antiviral treatment with VGCV on hearing and balance in children aged 6 months to 12 years. The ESPID group, however, has made clear that no consensus has been reached on how late it might be acceptable to start treatment for these patients, especially in the event of hearing deterioration. It therefore received a weak recommendation in favor of treatment (Quality D, Strength 2) in this group of patients. Despite its weak nature, this recommendation remains significant in that it applies to nearly half of the cCMV-related hearing loss cases in early childhood. Prompt testing for cCMV after failed newborn screening would be an effective means of avoiding any possible negative outcomes related to late treatment.

### 6.4. Benefit from Non-Pharmaceutical Treatment

Early hearing detection and intervention (EHDI) [72] efforts with a focus on hearing aid or cochlear implant use, as well as other forms of early intervention, may also benefit from selective cCMV screening. In the case of a sequential diagnostic paradigm, cCMV diagnostic testing following confirmation of congenital SNHL not only serves to abbreviate a potential diagnostic odyssey, but also appears cost-effective when compared to genetic testing or imaging [40]. The associated hearing aid fittings themselves are unlikely to be significantly affected by cCMV testing, excepting perhaps that, due to the often progressive nature of cCMV-related SNHL, the time periods between control appointments might need to be shortened. This heightened attention would allow for timely changes to hearing aid programming or a switch to more powerful hearing aids or even cochlear implants. In cases of severe hearing loss, even if unilateral or asymmetric, knowledge of the presence of cCMV infection might put cochlear implantation of the worse hearing ear up for discussion. This occurs as cCMV, due to its potential for causing progressive hearing loss over a period of years, might lead clinicians to consider early cochlear implantation of the worse hearing ear, as opposed to primarily orienting intervention and treatment plans to the better hearing ear [73,74,75,76].

The above considerations apply not only to both unilateral and bilateral cases of congenital SNHL, which to a great extent will be identified through selective CMV testing, but also to the other major part of cCMV-related SNHL. Specifically, it applies for newborns with delayed-onset SNHL with mostly asymptomatic, or at least not clinically diagnosable, CMV-infection who constitute about 46% (382 + 65/971) of the total disease burden (see Table 1). In asymptomatic cCMV infants that were identified through hospital-based newborn screening and were followed for a period of 18 years, Lanzieri et al. [77] recently reported that 25% developed SNHL. Of those ears, 65% exhibited progressive hearing loss bilaterally, such that 5% and 13% of participants were candidates for cochlear implantation in at least the worse hearing ear by 5 and 18 years of age, respectively. Considering this substantial burden of CMV-related SNHL in asymptomatic infants, with its potential impact on development, academic achievement and the need for ongoing audiological monitoring and interventions, SNHL, even if unilateral at first, can hardly be considered a “minimal deficit” [14]. Although the majority of those infants will be found through universal CMV screening, similar success can be anticipated for selective testing protocols at a greatly reduced cost, especially when NHS program referral rates are high.

## 7. Conclusions

Selective cCMV testing in newborns who fail newborn hearing screening allows for the identification of patients with congenital CMV that might otherwise be missed due to the lack of specific symptoms. However, PCR testing of urine or saliva needs to be performed within 3 weeks of age to allow for differentiation between congenital and postnatal CMV. Selective CMV testing can be estimated to identify at least 57% of congenital cCMV-related SNHL, but will nonetheless miss upwards of 43% of cases. This will most frequently occur in instances of mild hearing loss, though progressive and late-onset hearing loss have also proven difficult to identify consistently.

Two expert groups, the ICCRG [14] and the ESPID [15], unequivocally recommend antiviral therapy in newborns with moderate-to-severe symptomatic disease, as based on high level evidence from RCTs. Specifically, both guidelines interpret available data as suggesting that antiviral therapy prevents hearing deterioration in the short term and may prevent hearing deterioration at more than 1 year. Cases exhibiting moderate-to-severe symptoms comprise only a relatively minor proportion of cCMV, however, with the majority consisting of newborns with mild symptoms or isolated hearing loss, which by itself usually does not prompt CMV testing. For cases exhibiting mild symptomatology or isolated hearing loss, slightly diverging recommendations between the two groups can be seen to reflect controversies regarding the weighting of SNHL. Whereas the ICCRG recommend making decisions about antiviral treatment of isolated sensorineural hearing loss on a case-by-case basis, a majority of the ESPID expert group voted to include SNHL at birth in their indications for treatment, giving a strong recommendation, despite low overall quality of evidence.

The addition of isolated SNHL as an indication for antiviral treatment, irrespective of the presence or severity of additional clinical symptoms, results in a recommendation encompassing all cases of cCMV-related congenital SNHL. The potential benefit through antiviral therapy might thus extend to the 57% of cases with congenital SNHL detectable via selective testing. The remaining 43%, however, might only be detected via universal CMV screening. Though initial costs will necessarily rise due to the addition of the selective testing procedures, it remains that a dramatic improvement in the identification rate of asymptomatic newborns is possible, which not only results in reduced overall, long-term disease burden due to timely antiviral therapy as well as access to early intervention services, but also could do so at a financial cost far below that of a universal CMV screening program.

## Figures and Tables

**Table 1:** Numbers and proportions of cCMV-infected newborns per 1,000,000 births (with/without symptoms, not allowing/allowing clinical diagnosis, that will develop SNHL at birth or at late-onset up to 72 months of age) according to proportions calculated in [20].

Newborns	cCMV	Symptoms				Sensorineural Hearing Loss		
				Clinical Diagnosis		At Birth	Late Onset	Sum
1,000,000	6000		768		192	53	22	74
	0.6%	Yes	12.8%	Yes	25%	27.4%	11.2%	38.6%
					576	158	65	222
				No	75%	27.4%	11.2%	38.6%
			5232			293	382	675
		No	87.2%			5.6%	7.3%	12.9%
					Sum	503	468	971

## Abbreviations

CMV	cytomegalovirus
cCMV	congenital cytomegalovirus
DBS	dried blood spot
EHDI	early hearing detection and intervention
GCV	ganciclovir
PCHL	permanent childhood hearing loss
PCR	polymerase chain reaction
RCT	randomized controlled trial
SNHL	sensorineural hearing loss
UNHS	universal newborn hearing screening
VGCV	valganciclovir
